# Disruptive lysosomal-metabolic signaling and neurodevelopmental deficits that precede Purkinje cell loss in a mouse model of Niemann-Pick Type-C disease

**DOI:** 10.1038/s41598-023-32971-0

**Published:** 2023-04-06

**Authors:** Sarah Kim, Kathleen Ochoa, Sierra E. Melli, Fawad A. K. Yousufzai, Zerian D. Barrera, Aela A. Williams, Gianna McIntyre, Esteban Delgado, James N. Bolish, Collin M. Macleod, Mary Boghos, Hayden P. Lens, Alex G. Ramos, Vincent B. Wilson, Kelly Maloney, Zachary M. Padron, Amaal H. Khan, Rosa E. Blanco, Ileana Soto

**Affiliations:** 1 Department of Biology, Providence College, Providence, RI USA; 2 Department of Molecular and Cellular Biosciences, Rowan University, Glassboro, NJ USA; 3 Department of Chemistry and Biochemistry, Rowan University, Glassboro, NJ USA; 4 Department of Biological Science, Rowan University, Glassboro, NJ USA; 5 The Institute of Neurobiology, University of Puerto Rico, San Juan, PR USA

**Keywords:** Neurological disorders, Cell biology, Developmental biology, Neuroscience, Diseases

## Abstract

Purkinje cell (PC) loss occurs at an early age in patients and animal models of Niemann-Pick Type C (NPC), a lysosomal storage disease caused by mutations in the *Npc1* or *Npc2* genes. Although degeneration of PCs occurs early in NPC, little is known about how NPC1 deficiency affects the postnatal development of PCs. Using the *Npc1*^*nmf164*^ mouse model, we found that NPC1 deficiency significantly affected the postnatal development of PC dendrites and synapses. The developing dendrites of *Npc1*^*nmf164*^ PCs were significantly deficient in mitochondria and lysosomes. Furthermore, anabolic (mTORC1) and catabolic (TFEB) signaling pathways were not only perturbed but simultaneously activated in NPC1-deficient PCs, suggesting a loss of metabolic balance. We also found that mice with conditional heterozygous deletion of the Phosphatase and Tensin Homolog Deleted on Chromosome 10 gene (*Pten-*cHet), an inhibitor of mTORC1, showed similar early dendritic alterations in PCs to those found in *Npc1*-deficient mice. However, in contrast to *Npc1*^*nmf164*^ mice, *Pten-*cHet mice exhibited the overactivation of the mTORC1 pathway but with a strong inhibition of TFEB signaling, along with no dendritic mitochondrial reductions by the end of their postnatal development. Our data suggest that disruption of the lysosomal-metabolic signaling in PCs causes dendritic and synaptic developmental deficits that precede and promote their early degeneration in NPC.

## Introduction

NPC is an inherited lysosomal storage disease characterized by a neurovisceral accumulation of lipids that leads to neurodegeneration, ataxia, dementia, and death^1,2^. Although the onset of disease can occur at any age, the classic presentation of NPC is often found between middle to late childhood, when early symptoms such as clumsiness, vertical gaze palsy, gait disturbances, and eventually ataxia are evident^2^. Interestingly, cases of adult-onset NPC have been pre-diagnosed with mental disorders such as schizophrenia, attention deficit hyperactivity disorder (ADHD), and depression suggesting that neural circuitry dysfunction precedes the neurological symptoms^1,3^. Importantly, 80–90% of the NPC cases are caused by pathogenic variants in the *Npc1* gene^2^. NPC1 is a lysosomal and endosomal membrane protein that transports cholesterol out of these organelles. PCs are particularly susceptible to NPC1 deficiency by degenerating earlier and to a greater severity than other neurons in the brain^4^. Significant changes in cerebellar synapses and glial phenotypes along with behavioral and motor deficits are found during postnatal development and before PC degeneration^5–8^. However, little is known about how Npc1 deficiency affects PC development. To expand our understanding of how developmental deficits precede the dysfunction and degeneration of neurons in NPC, we investigated here how NPC1 deficiency in mice affects the postnatal development of PC dendrites.

It is known that mutations in *Npc1* produce the dysfunction of the proteolytic lysosomal pathway in cells^9,10^. Lysosomal dysfunction in NPC, and in other LSDs^11^, is concomitant with structural abnormalities and ectopic dendrites found in mature neurons from NPC patients and animal models^12,13^, suggesting that lysosomal dysfunction caused by *Npc1* deficiency modifies neuronal structure and function. Lysosomes have a central role in cellular metabolism^14^. In fact, the lack of lysosomal NPC1 causes the hyperactivation of the mTORC1 pathway, an evolutionarily conserved nutrient sensor that is the center of signaling networks that regulate cellular metabolic processes and growth^15^. Furthermore, hyperactivation of mTORC1 by NPC1 deficiency triggers lysosomal proteolytic dysfunction and mitochondria damage in cell culture models including neuronal cultures^9^, suggesting that lack of NPC1 disrupts cellular metabolism.

Because neuronal development is a complex and high-energy consuming process that involves the extension, patterning, and connectivity of neuronal processes^16^, we hypothesized that NPC1 deficiency leads to pathological changes in the postnatal development of PC dendrites by disrupting lysosomal-metabolic signaling. Given that mutations associated with mTORC1 regulation (e.g. *Pten*, *Tsc1*, *Tsc*2, *Pi3k*) lead to neurodevelopmental disorders like autism spectrum disorder^17^, it is imperative to understand the role of regulatory metabolic pathways in neurodevelopment. Using the late onset mouse model *Npc1*^*nmf164*^^18^, we investigated the effects of NPC1 deficiency in the postnatal development of PC dendrites and their metabolic signaling. The temporal and spatial activation of mTORC1 regulated proteins associated with anabolic and catabolic pathways were analyzed in healthy and *Npc1*^*nmf164*^ mouse PCs. Additionally, we performed similar analyses in mice with heterozygous conditional deletion of the *Pten* gene in PCs; PTEN is an endogenous inhibitor of mTORC1^19^. The *Pten-cHet* mice were used to differentiate the pathological changes induced by the disruption of mTORC1 from the pathology caused by lysosomal dysfunction, which is the case of the NPC disease.

## Results

### NPC1 deficiency disrupts postnatal development of Purkinje cell dendrites and synapses

**Figure 1:**
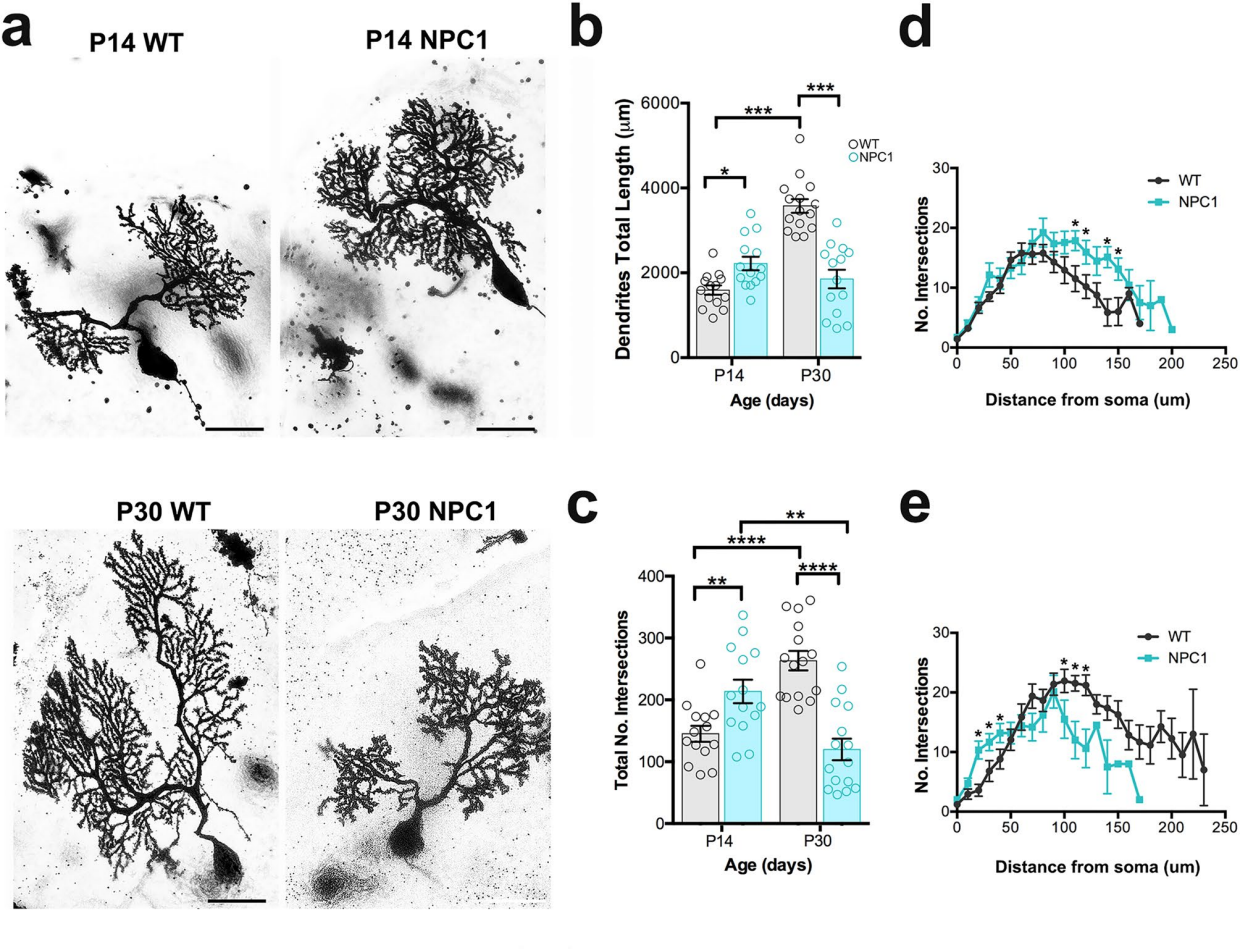
NPC1 deficiency alters the postnatal development of PC dendrites and synapses. (**a**) Golgi-Cox staining of PCs from WT and *Npc1*^*nmf164*^ (NPC1) mice at P14 and P30. (**b**) Measurement of dendrites total length from PCs at P14 and P30 showing significant differences between WT and *Npc1*^*nmf164*^ mice. Data are presented as mean ± SEM, n = 4 mice/genotype/age, 4–5 cells/mouse. (**c**) Measurement of total number of intersections calculated by Sholl analysis from PCs at P14 and P30 showing significant differences between WT and *Npc1*^*nmf164*^ mice. Data are presented as mean ± SEM, n = 4 mice/genotype/age, 4–5 cells/mouse. (**d**) Sholl-analysis showing differences in PC dendritic branching between WT and *Npc1*^*nmf164*^ mice at P14. (**e**) Sholl-analysis showing differences in PC dendritic branching between WT and *Npc1*^*nmf164*^ mice at P30. (**e**) Interactions between GFP^+^ PC dendritic spines and VGLUT1^+^ presynaptic terminals in WT and *Npc1*^*nmf164*^ mice at P14 and P21. Arrowheads showing VGLUT1^(+)^ GFP^+^ spines, arrows showing VGLUT1^(−)^ GFP^+^ spines. (**f**) Quantitative analysis of the total number of spines per μm. (**g**) Quantitative analysis of the number of spines that are contacted by VGLUT1^+^ presynaptic terminals. (**h**) Quantitative analysis of the number of spines that are not contacted (VGLUT1^(−)^) by VGLUT1^+^ presynaptic terminals. Data are presented as mean ± SEM, n = 3–4 mice/genotype/age, n = 10 dendritic segments/mouse. *P < 0.05, **P < 0.01, ***P < 0.001. Scale bar: (**a**) 20 μm (**e**) 3 μm.

Given that PC dendrites and synapses develop postnatally in mice, we examined how NPC1 deficiency alters PC dendrites at postnatal day 14 (P14) and 30 (P30) in *Npc1*^*nmf164*^ mice using the Golgi-Cox technique. Because PCs from the anterior lobules (I–V) have a similar developmental timeline^20^ and degenerate earlier than PCs in other cerebellar regions in NPC disease mouse models^7,21^, we have performed all the analyses in the first four lobules of the cerebellum. Quantitative analysis of PC dendrites showed a small but significant increase in the dendritic total length in *Npc1*^*nmf164*^ mice when compared to WT mice at P14 (Fig. 1a,b). In contrast, at P30, the dendritic total length was significantly reduced in *Npc1*^*nmf164*^ mice when compared to WT mice (Fig. 1a,b). As expected, the PC dendritic length in WT mice was significantly increased from P14 to P30, however, no differences in dendritic length between P14 and P30 were found in *Npc1*^*nmf164*^ mice (Fig. 1b). Similarly, the Sholl-analysis of PC dendrites indicated that *Npc1*^*nmf164*^ mice had a higher number of branches than WT mice at P14 (Fig. 1c,d), but a significant reduction of branches at P30 when compared to P30 WT mice and P14 *Npc1*^*nmf164*^ (Fig. 1c,e). These results suggest that deficiency of NPC1 causes an early and brief overgrowth of dendrites that is not sustained through the last two weeks of postnatal development but followed by dendritic regression. These changes in dendritic development were concomitant with similar changes in blood capillaries at the molecular layer (ML), which develop and grow along PC dendrites during postnatal development (Supplementary Fig. 1). The total length of Lectin immunoreactive capillaries and terminal points were significantly reduced in *Npc1*^*nmf164*^ mice at P30 than in WT mice (Supplementary Fig. 1).

**Figure 2:**
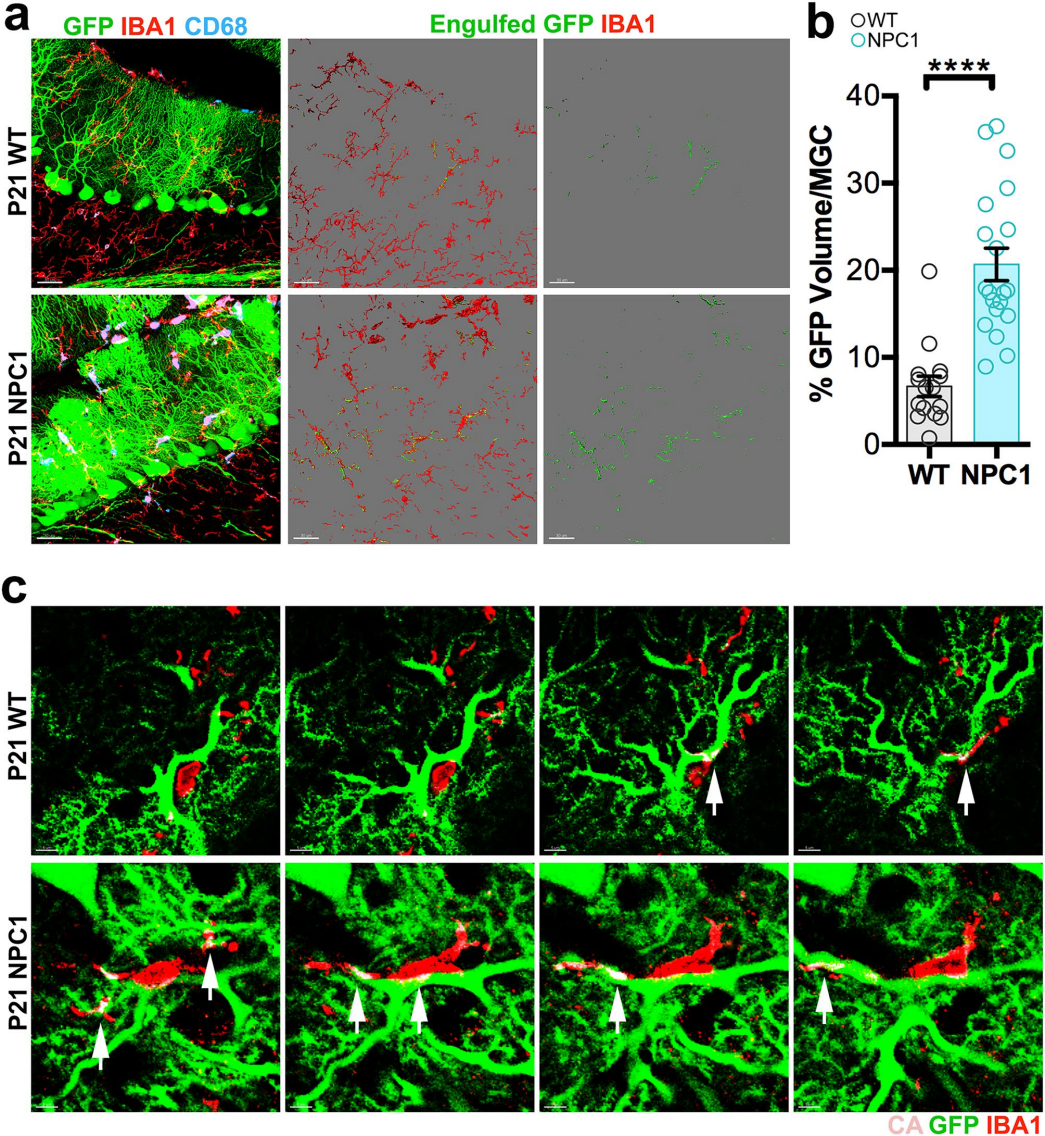
Increased contacts and engulfment of PC dendritic GFP and VGLUT1^+^ presynaptic inputs by microglia in P21 *Npc1*^*nmf164*^ mice. (**a**) Confocal images and 3D surface renderings showing segregated IBA1^+^ microglial cells from P21 WT and *Npc1*^*nmf164*^ mice contacting or engulfing GFP^+^ PC dendrites. The third column is showing 3D surface renderings of the contacted or engulfed GFP from PC dendrites. (**b**) Quantitative analysis of the total volume of contacted or engulfed GFP^+^ dendrites in P21 IBA1^+^ microglia. (**c**) High magnified serial sections from the confocal Z stack in (**a**) showing GFP^+^ dendrites contacted or engulfed (CA = contacted area, white arrows) by IBA1^+^ microglia at P21. Data are presented as mean ± SEM, WT n = 4–5 cells/mouse from 4 mice, *Npc1*^*nmf164*^ n = 4–5 cells from 4 mice. ****P < 0.0001. Scale bar: (**a**) 30 μm, (**c**) 5 μm.

Microglial cells in the ML are increasingly phagocytic in *Npc1*^*nmf164*^ mice at P14^5^. Since PC dendrites were significantly reduced in P30 *Npc1*^*nmf164*^ mice, using WT-*PCP2*^*EGFP*^ and *Npc1*^*nmf164*^*-PCP2*^*EGFP*^ mice we investigated microglia interactions with GFP^+^ dendrites. The interaction between IBA1^+^/CD68^+^ microglia and GFP^+^ PC dendrites was analyzed as previously reported^5,7^ using the 3D surface rendering tool from Imaris software (see “Methods”) to determine the percentage of GFP^+^ dendritic volume contacted or wrapped by microglia in *Npc1*^*nmf164*^ and WT mice. At P21, significantly increased volumes of GFP were found in *Npc1*^*nmf164*^ microglia when compared to microglia from WT mice (Fig. 2a–c), suggesting an increased interaction and engulfment of GFP^+^ dendrites by microglia in *Npc1*^*nmf164*^ mice.

**Figure 3:**
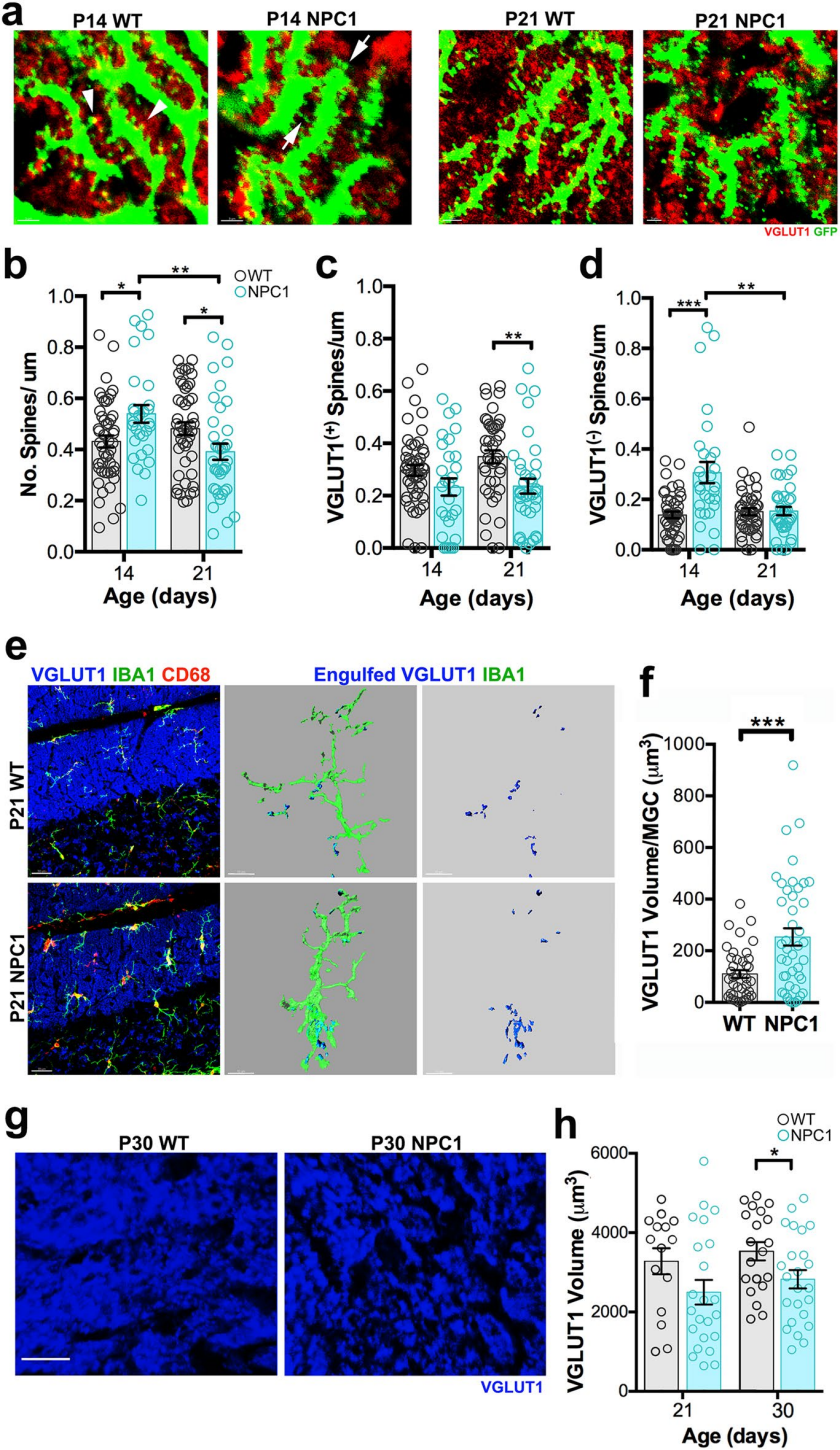
(**a**) Interactions between GFP^+^ PC dendritic spines and VGLUT1^+^ presynaptic terminals in WT and *Npc1*^*nmf164*^ mice at P14 and P21. Arrowheads showing VGLUT1^(+)^ GFP^+^ spines, arrows showing VGLUT1^(−)^ GFP^+^ spines. (**b**) Quantitative analysis of the total number of spines per μm. (**c**) Quantitative analysis of the number of spines that are contacted by VGLUT1^+^ presynaptic terminals. (**d**) Quantitative analysis of the number of spines that are not contacted (VGLUT1^(−)^) by VGLUT1^+^ presynaptic terminals. Data are presented as mean ± SEM, n = 3–4 mice/genotype/age, n = 10 dendritic segments/mouse. (**e**) Confocal images and 3D surface renderings showing segregated IBA1^+^ microglial cells from P21 WT and *Npc1*^*nmf164*^ mice contacting or engulfing VGLUT1^+^ presynaptic inputs. The third column is showing 3D surface renderings of the contacted or engulfed VGLUT1^+^ presynaptic inputs. (**f**) Quantitative analysis of the total volume of contacted or engulfed VGLUT1^+^ presynaptic inputs in P21 IBA1^+^ microglia. (**g**) Quantitative analysis of the total volume of VGLUT1^+^ presynaptic inputs in the ML of P30 mice. Data are presented as mean ± SEM, WT n = 44 cells from 4 mice, *Npc1*^*nmf164*^ n = 46 cells from 4 mice. *P < 0.05, **P < 0.01, ***P < 0.001, ****P < 0.0001. Scale bar: (**a**) 3 μm, (**e**) 30 μm, (**g**) 10 μm.

Because the formation of PC synapses with parallel fibers (PF) occurs along with the growth of dendrites and PC-PF developmental refinement falls between P14 and P30^22^, mice with GFP-expressing PCs were used to analyze dendritic spines colocalizing with VGLUT1^+^ PF inputs. WT-*PCP2*^*EGFP*^ and *Npc1*^*nmf164*^*-PCP2*^*EGFP*^ cerebellar sections immunolabeled with VGLUT1 were used to analyze synapses between GFP^+^ PC dendritic spines and VGLUT1^+^ PF presynaptic terminals. It is presumed that VGLUT1^(+)^-GFP^+^ PC spines are more mature than VGLUT1^(−)^-GFP^+^ PC spines because they are establishing synaptic contact with VGLUT1^(+)^ inputs. At P14 the total number of dendritic spines was significantly higher in *Npc1*^*nmf164*^ mice than in WT mice (Fig. 3a,b). However, most of these spines at P14 were not colocalizing with VGLUT1^+^ inputs (Fig. 3a,c), because the number of VGLUT1^(-)^-GFP^+^ PC spines was significantly higher in *Npc1*^*nmf164*^ mice than in WT mice (Fig. 3a,d). A significant reduction in the total number of spines was quantified in P21 *Npc1*^*nmf164*^ mice when compared to P14 *Npc1*^*nmf164*^ and P21 WT mice (Fig. 3a,b). While the number of VGLUT1^(+)^-GFP^+^ PC spines in P21 *Npc1*^*nmf164*^ mice was significantly decreased when compared to WT mice (Fig. 3c), a significant loss of VGLUT1^(−)^-GFP^+^ PC spines was found in P21 *Npc1*^*nmf164*^ when compared to P14 *Npc1*^*nmf164*^ (Fig. 3d). These data suggest that more spines in *Npc1*^*nmf164*^ mice were unable to establish connections with PF presynaptic terminals.

Given that microglia play important roles in developmental synapse elimination and refinement^23–25^, their potential involvement in the significant reduction of PF-PC synapses was investigated in developing *Npc1*^*nmf164*^ mice. We found that microglia were also engulfing significantly more VGLUT1^+^ PF presynaptic terminals in P21 *Npc1*^*nmf164*^ mice than in P21 WT mice (Fig. 3e–f). Although the volume of VGLUT1 immunoreactivity in the ML of *Npc1*^*nmf164*^ mice was slightly decreased at P21 and P30 than in WT mice, this reduction was only statistically significant at P30 (Fig. 3g,h). A previous study using *Npc1*^−/−^ mice found an imbalance between proliferation and differentiation of granule cells^26^; we found a small but significant increase in the volume of DAPI^+^ cells in the inner granule layer (IGL) in P30 *Npc1*^*nmf164*^ mice when compared to WT mice (Supplementary Fig. 2), suggesting that dysfunction of granule cells development as a potential contributor to changes in PF synapses cannot be completely discarded. Presynaptic terminals from CFs and GABAergic interneurons are also decreased in NPC1 deficient mice during postnatal development^5,6,27^, suggesting that PC dendritic deficits reported here may affect other processes of synaptic development. Overall, these findings suggest that after the first two weeks of postnatal development, *Npc1*^*nmf164*^ PCs are unable to continue growing and developing functional dendrites and synapses leading to the loss and increased interaction of these structures with microglia.

### NPC1-deficient Purkinje cells have reduced mitochondrial and lysosomal volume in dendrites

**Figure 4:**
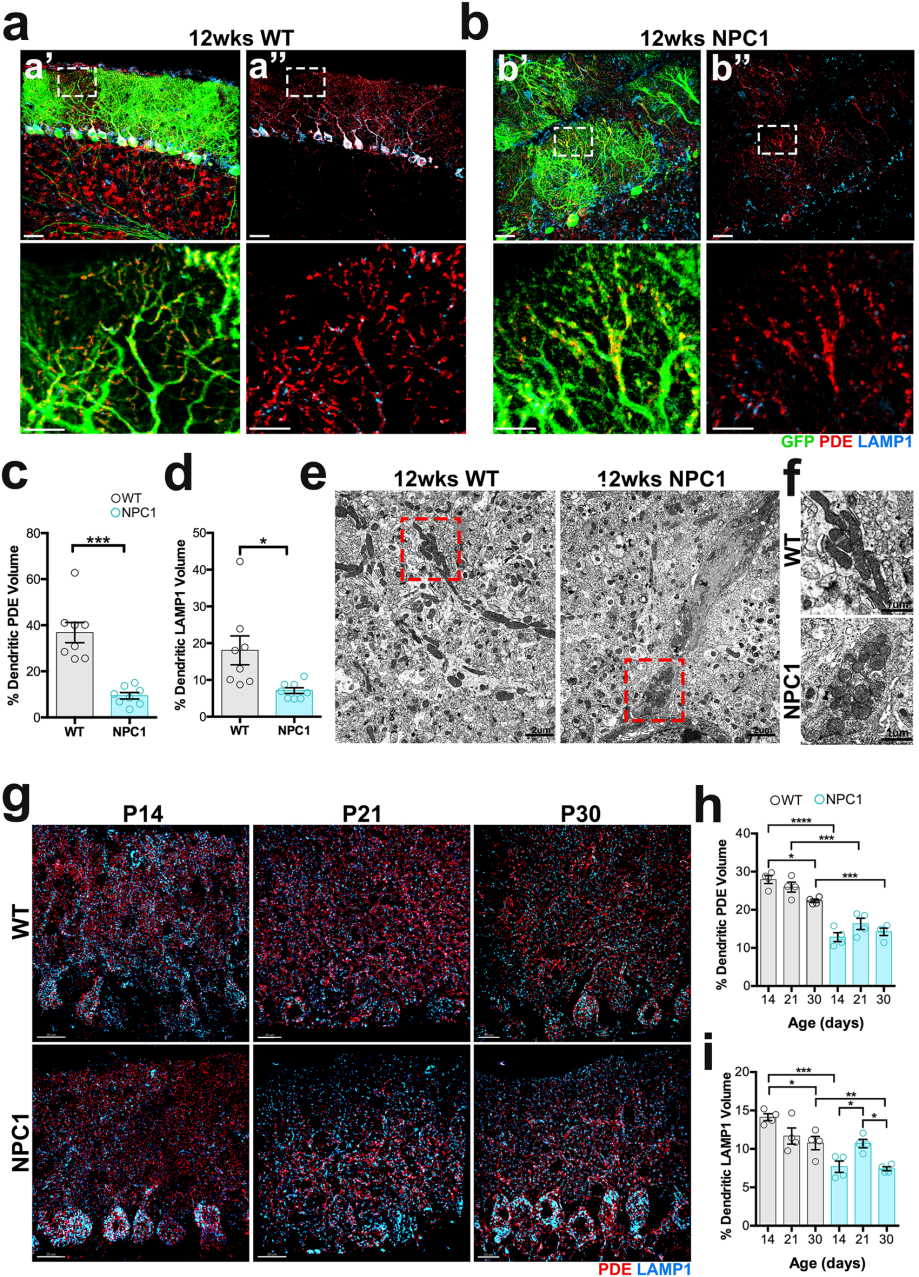
NPC1 deficiency decreases mitochondrial and lysosomal levels in PC dendrites during neurodegeneration and postnatal developmental stages. (**a**,**b**) Images of 12wks cerebellar sections showing the GFP^+^ PCs in WT (**a**) and *Npc1*^*nmf164*^ (**b**) mice immunolabeled with antibodies against PDE and LAMP (**a′** and **b′**). Fluorescent signal from PDE and LAMP1 only in PCs was segregated using the Imaris software for analysis (**a″** and **b″**). High magnified images from insets in (**a**,**b**) show dendritic mitochondria density and distribution. (**c**) Quantification of the percentage of PC dendritic PDE volume in WT and *Npc1*^*nmf164*^ mice. (**d**) Quantification of the percentage of PC dendritic LAMP1 volume in WT and *Npc1*^*nmf164*^ mice. Data are presented as mean ± SEM, n = 4 mice/genotype, 2 images/mouse. *P < 0.05, **P < 0.001, ***P < 0.001, ****P < 0.0001. (**e**) Transmission electron micrographs showing PCs dendrites segments containing mitochondria with significant differences in distribution and morphology between WT and *Npc1*^*nmf164*^ mice. (**f**) Insets are magnified images from (**e**) showing mitochondria morphology and aggregation in *Npc1*^*nmf164*^ mice. (**g**) Segregated PDE (mitochondria) and LAMP1 (lysosomes) in PCs using Imaris software in WT and *Npc1*^*nmf164*^ (NPC1) mice at P14, P21, and P30. (**h**) Measurement of the percentage of PDE volume in PCs dendrites at P14, P21, and P30 showing significant differences between WT and *Npc1*^*nmf164*^ mice. (**i**) Measurement of the percentage of LAMP1 volume in PCs dendrites at P14, P21, and P30 showing significant differences between WT and *Npc1*^*nmf164*^ mice. Data are presented as mean ± SEM, n = 4 mice/genotype. *P < 0.05, **P < 0.001, ***P < 0.001, ****P < 0.0001. Scale bar: (**a**,**b**) 30 μm and inset 10 μm, (**e**) 2 μm, (**f**) 1 μm, (**g**) 20 μm.

Recent studies have shown that NPC1 deficiency causes lysosomal dysfunction and mitochondrial damage in vitro^9,28^, but not in vivo analysis of mitochondria in NPC models has been reported. Here, using the Imaris 3D surface rendering tool we were able to select and measure the total volume of PDE (pyruvate dehydrogenase E 1 alpha) immunoreactive mitochondria and LAMP1 immunoreactive lysosomes only in CALB immunoreactive PCs in mice (Supplementary Fig. 3). First, we examined the levels of mitochondrial and lysosomal volume in *Npc1*^*nmf164*^*-Pcp2*^*EGF*^ mice at age 12 weeks when severe degeneration of PCs occurs^7^, and found that at this stage of neurodegeneration, the percentage of mitochondrial and lysosomal volume in PC dendrites from *Npc1*^*nmf164*^ mice was significantly reduced when compared to WT mice (Fig. 4a–d). Additionally, mitochondria in *Npc1*^*nmf164*^ dendrites were not only fragmented but also aggregated in swollen dendritic portions of NPC1-deficient PC dendrites (Fig. 4b). These findings were also confirmed using transmission electron microscopy (TEM), where healthy elongated mitochondria were observed in PC dendrites from 12 weeks WT mice, while fragmented, rounded, and aggregated mitochondria were observed in swollen degenerating 12 weeks *Npc1*^*nmf164*^ dendrites (Fig. 4e,f). These results support the hypothesis that impaired lysosomal function in NPC leads to the aggregation of damaged mitochondria, metabolic deficits, and cell death. Therefore, we examined the levels of PDE^+^ mitochondrial and LAMP1^+^ lysosomal volume in PC dendrites at different stages of postnatal development in *Npc1*^*nmf164*^ and WT mice. The percentage of mitochondria volume in PC dendrites of WT mice was slightly decreased between P14 and P30 (Fig. 4g,h), which could be the result of existent mitochondria at P14 becoming sparser throughout larger dendritic trees at P30. However, the percentage of mitochondria volume in *Npc1*^*nmf164*^ PC dendrites was significantly lower at P14, P21, and P30 than in WT (Fig. 4g,h). Levels of PDE protein were found reduced in P30 *Npc1*^*nmf164*^ mice by western blot but did not reach statistical significance (Supplementary Fig. 4). In the case of lysosomes, when compared to WT mice, the percentage of LAMP1 + lysosomal volume in PC dendrites from *Npc1*^nmf164^ mice was lesser at P14 and P30, but the same at P21(Fig. 4g,i). This suggests that at P21 a brief increase of lysosomes occurs in NPC1-deficient PCs. Our data suggest that during development, lysosomal dysfunction causes mitochondrial deficits in PC dendrites that precede and persist through the degeneration of PCs in NPC.

### Disruptive lysosomal-metabolic signaling in developmental ***Npc1***^***nmf164***^ Purkinje cells

**Figure 5:**
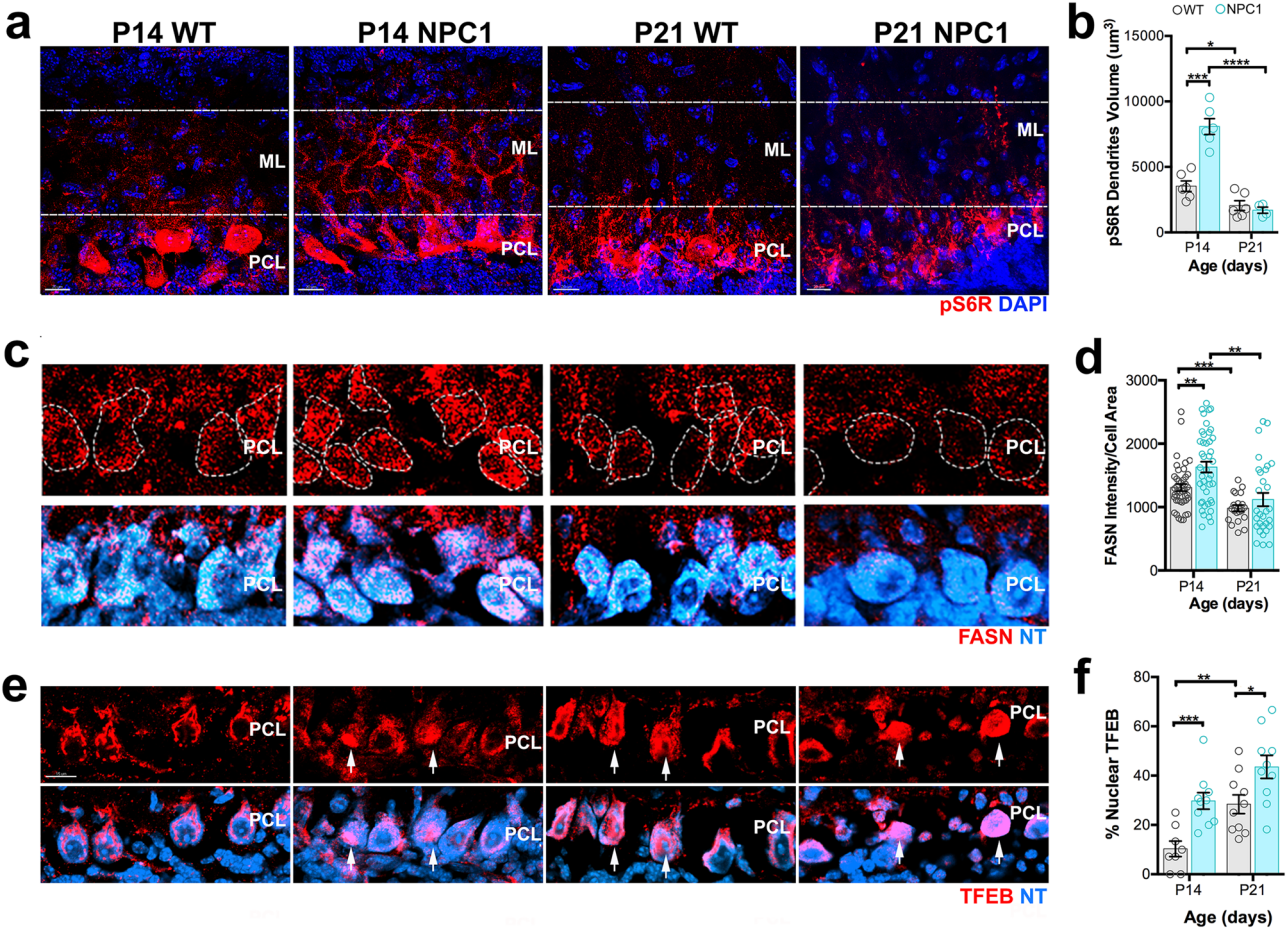
Simultaneous overactivation of the mTORC1 pathway and TFEB occurs in PCs from P14 *Npc1*^*nmf164*^ mice. (**a**) Confocal images showing pS6R immunoreactivity in the cerebellar PCL and ML of P14 and P21 WT and *Npc1*^*nmf164*^ mice. Dotted lines indicate the region used for quantitative analysis. (**b**) Quantitative analysis of the total volume of pS6R in PC dendrites from WT and *Npc1*^*nmf164*^ mice at P14 and P21. Data are presented as mean ± SEM, n = 3 mice/genotype/age, 2 images/mouse. (**c**) Confocal images showing FASN immunoreactivity and NeuroTrace (NT) staining in the cerebellar PCL of P14 and P21 WT and *Npc1*^*nmf164*^ mice. (**d**) Quantitative analysis of FASN fluorescence intensity in NT^+^ PCs from WT and *Npc1*^*nmf164*^ mice at P14 and P21. Data are presented as mean ± SEM, n = 10 cells/mouse from 4 mice/genotype/age. (**e**) TFEB immunoreactivity in PCs from WT and *Npc1*^*nmf164*^ mice at P14 and P21; nuclear localization of TFEB is indicated by white arrows. (**f**) Percentage of PCs showing nuclear translocation of TFEB in WT and *Npc1*^*nmf164*^ mice at P14 and P21. Data are presented as mean ± SEM, n = 4 mice/genotype/age, 2–3 images/mouse. *P < 0.05, **P < 0.001, ***P < 0.001, ****P < 0.0001. Scale bars: (**a**) and (**c**) 20 μm, (**e**) 15 μm.

It is well known that lysosomal cholesterol accumulation caused by NPC1 deficiency in different in vitro cell culture models leads to the hyperactivation of the anabolic mTORC1 pathway^9,28^. It was intriguing to find that the size of the PCs dendritic tree and the number of spines were increased in P14 *Npc1*^*nmf164*^ mice when compared to WT. We hypothesized that this increase was the result of mTORC1 hyperactivation. To test this, we used an antibody against a specific downstream target of the mTORC1 pathway, the phosphorylated S6 ribosomal (pS6R) protein. Strong immunolabeling of pS6R was observed in somas of PCs from *Npc1*^*nmf164*^ and WT mice (Fig. 5a). However, pS6R immunoreactivity was significantly higher in PC dendrites from P14 *Npc1*^*nmf164*^ mice (Supplementary Fig. 5) when compared to P14 WT mice (Fig. 5a,b). Additionally, P14 WT mice showed higher levels of dendritic pS6R immunoreactivity when compared to P21 WT mice. These data suggest that activation of mTORC1 occurs in dendrites during stages of active growth (P14), but NPC1 deficiency induces a further increase of this basal activation that could be contributing to the increased growth of dendrites in P14 *Npc1*^*nmf164*^ mice.

Since the mTORC1 pathway is associated with the activation of anabolic pathways such as lipid biogenesis^29^, we investigated the levels of the fatty acid synthase (FASN) protein in PCs using immunofluorescence. Transcription of FASN is regulated by the sterol regulatory element binding protein 1 (SREBP1) transcription factor^30,31^, which can be activated by the mTORC1 signaling^29,32,33^. Quantitative analysis of FASN fluorescence intensity in PC somas showed that FASN levels were higher in P14 WT mice than in P21 WT mice (Fig. 5c,d). PCs from P14 *Npc1*^*nmf164*^ mice showed higher levels of FASN when compared to PCs from P14 WT and P21 *Npc1*^*nmf164*^ mice (Fig. 5c,d). This suggests that, like mTORC1, FASN levels are increased in PCs during stages of dendritic growth and that hyperactivation of mTORC1 in *Npc1*^*nmf164*^ PCs may be leading to the heightened increase of FASN in these mice.

Finally, because activation of the mTORC1 pathway leads to the blockage of the transcription factor EB (TFEB), which upon activation induces biogenesis of lysosomes and catabolic pathways^34,35^, we examined the activation of TFEB in PCs using immunofluorescence. The percentage of PCs with nuclear localization of TFEB, a well-established hallmark of TFEB activation^36^, was determined in WT and *Npc1*^*nmf164*^ mice at postnatal stages. Our data showed that the percentage of nuclear TFEB in PCs while low at P14 in WT PCs was significantly increased by P21 (Fig. 5e,f). However, in *Npc1*^*nmf164*^ mice, the levels of nuclear TFEB were significantly higher at P14 than in P14 WT, and even higher than P21 WT in P21 mice. It was surprising to find increased levels of TFEB in P14 *Npc1*^*nmf164*^ PCs because the mTORC1pathway was hyperactivated at this stage. However, it is possible that the decreased levels and dysfunction of lysosomes in NPC1-deficient PCs led to the activation of other upstream pathways that can activate TFEB to compensate for the deficiency. To test this, we proceeded to analyze PCs from PTEN conditional heterozygous (*Pten-*cHet) mice where the mTORC1 pathway can be hyperactivated specifically in PCs that have healthy lysosomes.

### *Pten* conditional heterozygosity in Purkinje cells alters their dendritic development

**Figure 6:**
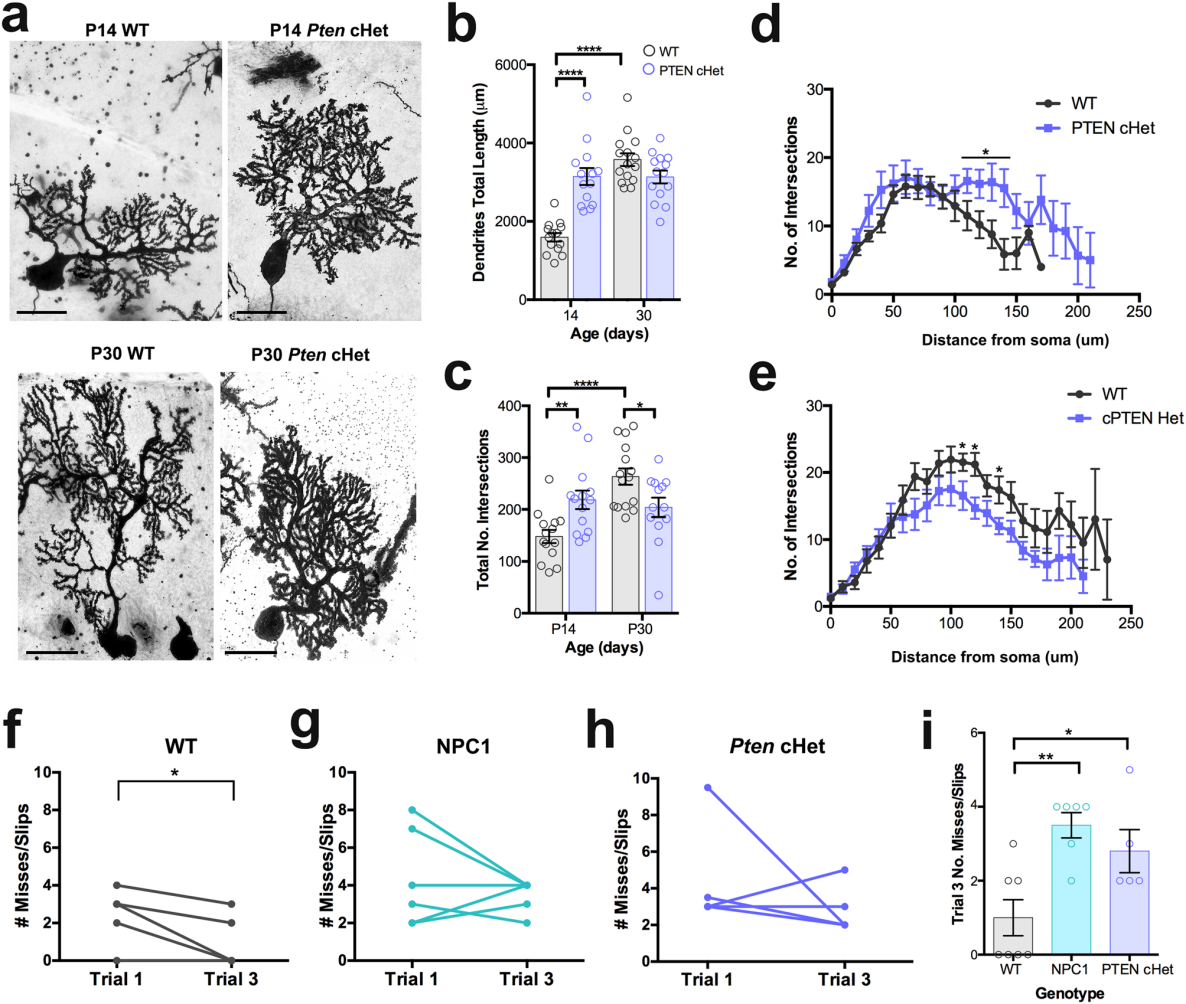
*Pten* conditional haploinsufficiency in PCs alters the postnatal development of their dendrites at P14. (**a**) Golgi-Cox staining of PCs from WT and *Pten-*cHet mice at P14 and P30. (**b**) Quantification of PC dendritic total length at P14 and P30 showing significant differences between WT and *Pten-*cHet mice at P14, no changes were found at P30. (**c**) Quantification of dendritic total intersections calculated by Sholl analysis at P14 and P30 showing significant differences between WT and *Pten-*cHet mice. Data in (**a**) and (**b**) are presented as mean ± SEM, n = 4 mice/genotype/age, 4–5 cells/mouse. (**d**) Sholl-analysis showing differences in PC dendritic branching between WT and *Pten-*cHet mice at P14. (**e**) Sholl-analysis showing differences in PC dendritic branching between WT and *Pten-*cHet mice at P30. Data are presented as mean ± SEM, n = 4 mice/genotype/age, 4–5 cells/mouse. ***P < 0.001, ****P < 0.0001. (**f**) The number of mises/slips in the ladder rung task were significantly decreased between trials 1 and 3 in P30 WT mice (n = 7). (**g**,**h**) No significant differences in number of mises/slips in the ladder rung task were found between trials 1 and 3 in P30 *Npc1*^*nmf164*^ (**g**, n = 6) and *Pten-*cHet (**h**, n = 5) mice. (**i**) The total number of misses/slips in trial 3 was significantly higher in *Npc1*^*nmf164*^ (n = 6) and *Pten-*cHet (n = 5) mice when compared to WT mice (n = 7). Data are presented as mean ± SEM, n = 2 images from 4 mice/genotype/age. *P < 0.05, **P < 0.001. Scale bar: (**a**) 20 μm.

Previous studies demonstrated that PC conditional deletion of PTEN leads to cell hypertrophy, age-dependent degeneration, and behavioral deficits^37^. Interestingly, *Pten* heterozygous mutations in humans and animal models are associated with autism spectrum disorder, macrocephaly, and neuronal hypertrophy including PCs^38,39^. To test if PTEN haploinsufficiency alters PC dendritic development, conditional heterozygous mice for the *Pten* gene (*Pten-*cHet) in PCs were used to examine changes in dendritic development at P14 and P30. Using the Golgi-Cox technique, we found that the dendritic total length and branching in *Pten-*cHet mice were significantly higher in P14 than in P14 WT mice (Fig. 6a–d). At P30 however, there was no difference in the total length of dendritic trees between *Pten-*cHet and WT mice (Fig. 6b), but the branching, as measured by the number of intersections by Sholl analysis, was significantly decreased in *Pten-*cHet (Fig. 6c,e). These data suggest that *Pten* haploinsufficiency produces dendritic overgrowth only during the first two weeks of postnatal development followed by a lack of growth during the last two weeks of postnatal development.

To test if the pathological changes in dendritic development observed in *Npc1*^*nmf164*^ and *Pten-*cHet PCs lead to motor deficits, the ladder rung walking task^40^ was performed in WT and both mutant mouse strains. The number of misses and slips, including the failure to put the paw directly onto the rung, placing of the paws between the rungs, or paws slipping off the rungs, was calculated. Mice were allowed to walk through the horizontal ladder rung three times, while expecting the decrease of misses and slips by the third trial. Though P30 WT mice were able to significantly improve their motor coordination through the horizontal ladder by trial 3 when compared to trial 1 (Fig. 6f), P30 *Npc1*^*nmf164*^ and *Pten-*cHet mice were unable to show improvements between trials and had a significant increased number of misses and slips at trial 3 when compared to WT mice (Fig. 6g–i). These findings demonstrate that dendritic neurodevelopmental defects in PCs lead to motor deficits in *Npc1*^*nmf164*^ and *Pten-*cHet mice.

### Hyperactivation of the AKT-mTORC1 pathway in *Pten* conditional heterozygous Purkinje cells

**Figure 7:**
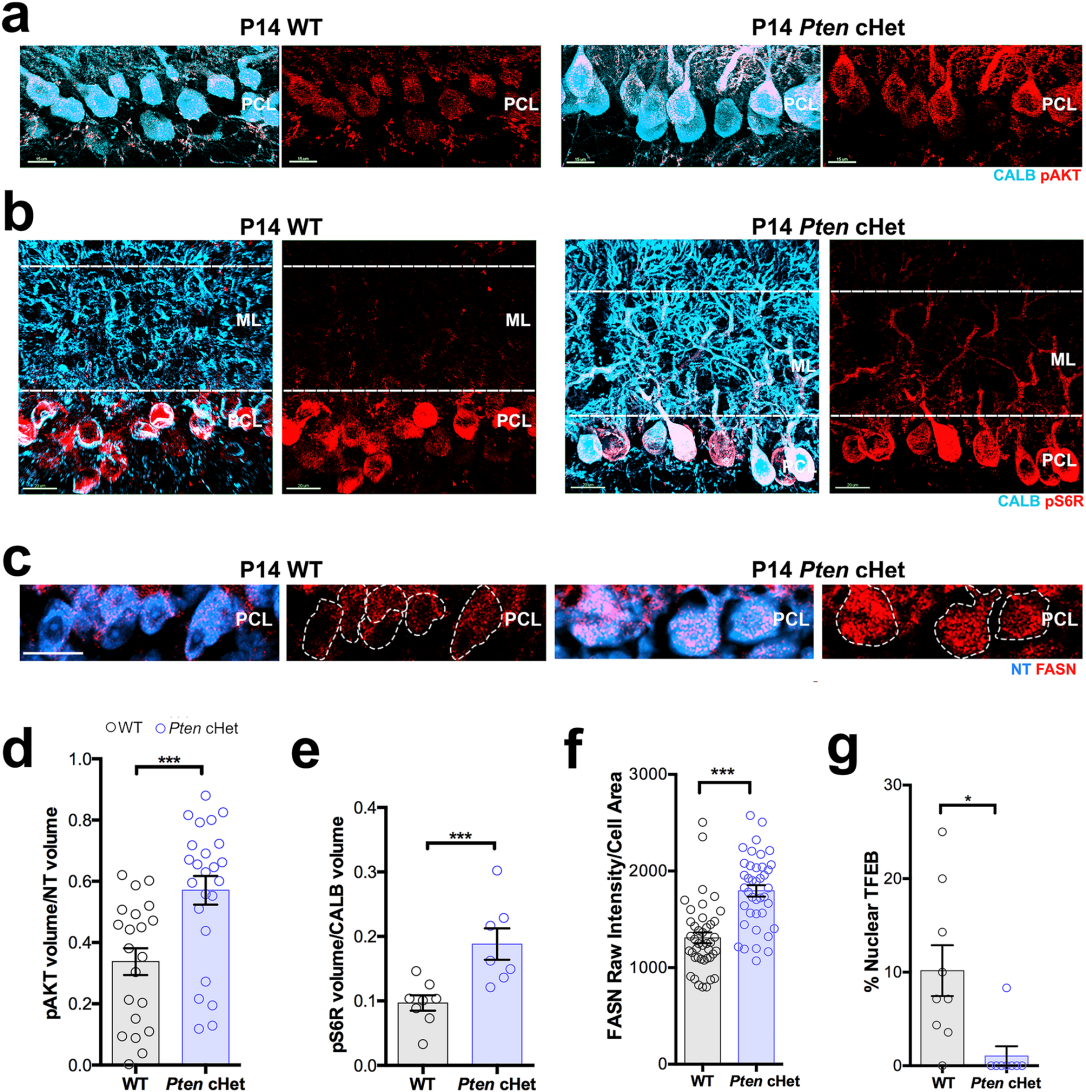
Overactivation of the AKT-mTORC1 pathway in *Pten-*cHet PCs from P14 mice. (**a**) Confocal images showing pAKT and CALB immunoreactivity in P14 PCs from WT and *Pten-*cHet mice. (**b**) Confocal images showing pS6R and CALB immunoreactivity in the cerebellar PCL and ML of P14 WT and *Pten-*cHet mice. Dotted lines indicate the region used for quantitative analysis. (**c**) Confocal images showing FASN immunoreactivity and NT staining in the cerebellar PCL of P14 WT and *Pten-*cHet mice. (**d**) Quantitative analysis of the ratio of pAKT volume in CALB^+^ PC soma from WT and *Pten-*cHet mice at P14. Data are presented as mean ± SEM, n = 4 mice/genotype/age, 5–6 cells/mouse. (**e**) Quantitative analysis of the ratio of pS6R volume per dendritic CALB volume from WT and *Pten-*cHet mice at P14. Data are presented as mean ± SEM, n = 4 mice/genotype/age, 2 images/mouse. (**f**) Quantitative analysis of FASN fluorescence intensity in NT^+^ PCs from WT and *Pten-*cHet mice at P14. Data are presented as mean ± SEM, n = 10 cells from 4 mice/genotype/age. (**g**) Quantitative analysis of the percentage of PCs with nuclear TFEB from WT and *Pten-*cHet mice at P14. Data are presented as mean ± SEM, n = 2 images from 4 mice/genotype/age. ***P < 0.001. Scale bars: (**a**) 15 μm, (**b**) and (**c**) 20 μm.

PTEN is a well-known endogenous inhibitor of the AKT-mTORC1 pathway^19^. To determine if *Pten* haploinsufficiency in PCs induces the hyperactivation of AKT, PCs immunoreacted with phosphorylated AKT (pAKT) and CALB were analyzed at P14 in *Pten-*cHet and WT mice. We found that pAKT was significantly higher in P14 *Pten-*cHet PC somas than in WT (Fig. 7a,d), confirming that *Pten* haploinsufficiency causes the hyperactivation of AKT in PCs. We also found that the pS6R volume in CALB^+^ PCs was significantly greater in dendrites from *Pten-*cHet mice than in WT mice (Fig. 7b,e). Similarly, the immunoreactivity of the mTORC1 downstream protein FASN was found to be significantly higher in PC somas from *Pten-*cHet mice than in WT mice (Fig. 7c,f), suggesting that haploinsufficiency of *Pten* in P14 mice leads to the hyperactivation of the AKT-mTORC1 pathway, increased lipid biogenesis, and overgrowth of PC dendrites in these mice. As expected, nuclear translocation of TFEB, which is inhibited by both AKT and mTORC1, was significantly lower (almost none) in *Pten-*cHet PCs when compared to WT mice (Fig. 7g).

**Figure 8:**
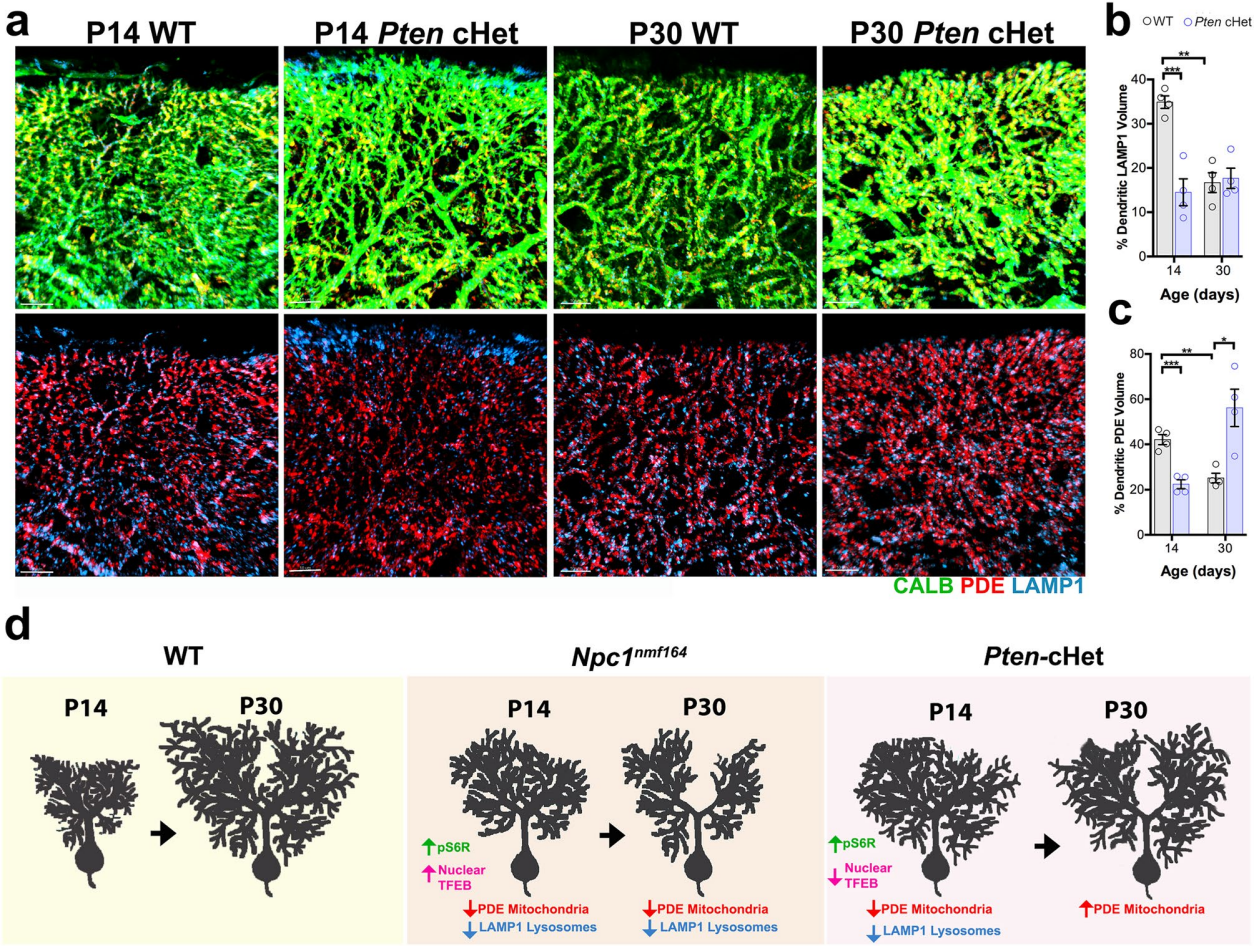
*Pten* conditional haploinsufficiency in PCs alters mitochondrial and lysosomal levels in PC dendrites during postnatal development. (**a**) Segregated PDE (mitochondria) and LAMP1 (lysosomes) immunoreactivity in CALB^+^ PC dendrites in WT and *Pten-*cHet mice at P14 and P30. (**b**) Measurement of the percentage of LAMP1 volume in PCs dendrites at P14 and P30 showing significant differences between WT and *Pten-*cHet mice. (**c**) Measurement of the percentage of PDE volume in PCs dendrites at P14 and P30 showing significant differences between WT and *Pten-*cHet mice. Data are presented as mean ± SEM, n = 4 mice/genotype/age. **P < 0.001, ***P < 0.001. Scale bars: (**a**) 5 μm. (**d**) Schematic illustration showing how changes in activation of the mTORC1 pathway (pS6R) and TFEB occur along with significant changes in the size of the dendritic tree of PCs from *Npc1*^*nmf164*^ and *Pten-*cHet mice during postnatal development. Changes in dendritic mitochondrial and lysosomal volume were evident in the different mouse strains, suggesting that lysosomal dysfunction in NPC1 deficient mice disturbs metabolic balance and causes dendritic atrophy.

Contrary to *Npc1*^*nmf164*^ mice, *Pten-*cHet mice should have healthy and functional lysosomes. However, it is expected that due to the hyperactivation of the AKT-mTORC1 pathway and the lack of activation of TFEB, the biogenesis of lysosomes may be decreased in the PCs of these mice. After examining P14 and P30 *Pten-*cHet mice we found significantly decreased percentage of LAMP1^+^ lysosomal volume in CALB^+^ PC dendrites in P14 *Pten-*cHet mice when compared to WT (Fig. 8a,b). To determine if overactivation of the AKT-mTORC1 pathway in *Pten-*cHet mice leads to changes in mitochondria, the percentage of PDE^+^ mitochondrial volume was measured in CALB^+^ PCs dendrites. Our results showed that the percentage of mitochondrial volume in PC dendrites at P14 was significantly decreased in *Pten-*cHet mice than in WT (Fig. 8a–c). Western blot analysis of PDE protein levels in *Pten-*cHet cerebella at this postnatal stage (P14) showed no significant differences when compared to P14 WT cerebella (Supplementary Fig. 5), suggesting that the levels of mitochondria in *Pten-*cHet PCs were not increased in response to dendritic overgrowth. Conversely, at P30 the percentage of mitochondria volume in PC dendrites was significantly increased in *Pten-*cHet mice when compared to WT mice (Fig. 8a–c). These results suggest that mitochondria biogenesis in *Pten-*cHet mice may increase to compensate for the early overgrowth of dendrites even when no further overgrowth of dendrites occurred after the first two postnatal weeks.

Overall, these findings have shown that early dendritic overgrowth and overactivation of the mTORC1 pathway (pS6R) in PCs occur in both *Npc1*^*nmf164*^ and *Pten-*cHet mouse strains (Fig. 8d). However, simultaneous and persistent overactivation of TFEB and decreased levels of mitochondrial volume were only found in *Npc1*^*nmf164*^ PCs where significant dendritic atrophy occurs by the end of postnatal development (Fig. 8d).

## Discussion

NPC patients are still developing their brains at the time of disease onset; therefore, we were intrigued by how lack of NPC1 impacts neurodevelopment and predisposes neurons to early degeneration. Importantly, the development of early neurological symptoms such as clumsiness, gait defects, and ataxia in both the human disease and animal models are a direct consequence of early dysfunction and degeneration of cerebellar PCs^2^. However, little is known about how NPC1 deficiency affects PCs development and if neurodevelopmental deficits precede their precipitated death. Given the abrupt early loss of PCs in NPC during childhood and juvenile ages, we hypothesized that in the *Npc1*^*nmf164*^ mouse postnatal development, alterations of PCs precede and predispose these neurons to cell death. Here, we showed that not only NPC1 deficiency impacts the development and metabolic signaling of PC dendrites, but also has contrasting effects through different stages of postnatal development. For instance, a small but significant increase in the size of PCs dendritic tree in P14 *Npc1*^*nmf164*^ mice was concomitant with increased levels of pS6R and FASN, both downstream products of mTORC1 activation^32^. Hyperactivation of the mTORC1 pathway due to NPC1 deficiency occurs in several cell culture models including iPSC-derived neurons^9,41^. Similarly, higher levels of pS6R and FASN were found in PCs from *Pten-*cHet mice, where a major overgrowth of PC dendrites was also found at P14. Increased levels of the FASN protein are a hallmark of increased activation of the SREBP1 transcription factor, which regulates the expression of genes that control lipid biogenesis and homeostasis^42^. Regulation of the SREBP transcription factors by the mTORC1 pathway contributes to cell growth^29,32,33^. SREBPs and the activation of fatty acid synthesis play important roles in the dendritic development of neurons of *Drosophila* larvae^43,44^ and mouse hippocampal neurons^45^. Our results suggest that hyperactivation of mTORC1 in PCs leads to the increased activation of anabolic pathways as demonstrated by the increased phosphorylation of S6R and synthesis of the FASN protein in P14 *Npc1*^*nmf164*^ and *Pten-*cHet PCs. Further studies are necessary to confirm if the overactivation of SREBP1 transcription factor in PCs is directly caused by the hyperactivation of the mTORC1 pathway.

Structural abnormalities and ectopic dendrites are found in neurons from NPC patients, and in mouse and cat animal models^12,13^, suggesting that lysosomal dysfunction impacts neuronal structure and function. Our results are in line with these findings as NPC1 deficiency yielded significant changes in the development of PC dendrites and synaptic structures. In fact, the overgrowth of dendrites and spines found in *Npc1*^*nmf164*^ PCs at P14 was not sustained through the postnatal third and fourth week. Moreover, the dendritic trees of *Npc1*^nmf164^ PCs were significantly reduced at P30 when compared to WT mice. These results coincided with the lack of hyperactivation of the S6R protein and the stabilization of the FASN protein levels at P21 in *Npc1*^*nmf164*^ PCs, both proteins levels were similar to WT. Overactivation of anabolic processes during growth could lead to states of nutrient starvation and low energy, leading to inhibition of mTORC1^46^. However, while the levels of these mTORC1 downstream proteins were similar between *Npc1*^nmf164^ and WT mice at P21, at P30 the size of the PC dendritic trees in *Npc1*^nmf164^ were still significantly smaller than WT PC dendritic trees. Furthermore, in P14 *Pten-*cHet mice, PC dendritic trees were not as reduced as in the *Npc1*^*nmf164*^ mice. These results suggested that other pathological changes associated with lysosomal dysfunction in *Npc1*^*nmf164*^ mice were driving the remarkable dendritic atrophy at P30. Given that mTORC1 activation leads to the inhibition of TFEB nuclear translocation and activation, decreased nuclear translocation of TFEB was expected in P14 *Npc1*^*nmf164*^ mice, but nuclear translocation of TFEB was significantly increased in P14 and P21 *Npc1*^*nmf164*^ PCs. Interestingly, at P21 decreased pS6R levels coincided with increased nuclear translocation of TFEB in WT PCs, suggesting that activation of catabolic pathways at this age may be associated with the process of dendritic remodeling (from multiplanar to monoplanar) that occurs in these cells between P18 and P25^47^. As anticipated, and contrary to the findings in *Npc1*^*nmf164*^ mice, *Pten-*cHet PCs exhibited a significant decrease in TFEB nuclear translocation. *Pten* haploinsufficiency is expected to induce the overactivation of the AKT-mTORC1 pathway; both AKT^48,49^ and mTORC1^50,51^ can inhibit TFEB nuclear translocation leading to the inhibition of catabolic pathways. It is possible that lysosomal dysfunction in NPC not only causes the aberrant overactivation of the mTORC1 pathway, but also the co-activation of other molecular pathways that respond to lysosomal deficits. One possible mechanism driven by lysosomal dysfunction to co-activate TFEB in *Npc1*^*nmf164*^ mice is the deregulation of intracellular Ca^2+^. NPC1-deficient cells have decreased levels of lysosomal and endoplasmic reticulum (ER)-luminal Ca^2+^ and increased influx of Ca^2+^ at the plasma membrane resulting in an increased resting cytosolic Ca^2+^^52–54^. This increased intracellular Ca^2+^ can activate cytoplasmic calcineurin, a phosphatase that induces TFEB nuclear translocation after dephosphorylation^55–57^, suggesting that the aberrant activation of TFEB in *Npc1*^*nmf164*^ PCs may be the consequence of deregulation of the Ca^2+^ signaling. Future studies are needed to determine if Ca^2+^ deregulation in NPC leads to the aberrant activation of TFEB in developing PCs.

Another potential trigger of TFEB activation is the accumulation of damaged mitochondria^58^. The biogenesis of mitochondria is significantly decreased in NPC1-deficient cells due to lysosomal dysfunction^59^, and the accumulation of damaged mitochondria in these cells is a direct consequence of mTORC1 hyperactivation^9^. Our results suggest that in *Npc1*^*nmf164*^ mice, the significant deficiency of healthy mitochondria and the accumulation of the damaged type could also be triggers of TFEB overactivation. First, we found accumulation of damaged mitochondria in atrophied dendrites from degenerating PCs in 12 weeks *Npc1*^*nmf164*^ mice. Levels of PDE^+^ mitochondria in PC dendrites were significantly reduced through all the analyzed postnatal stages, supporting the idea that PC mitochondria deficits are an early event that precedes neurodegeneration in NPC. Given that lysosomes and mitochondria are important mediators of cellular metabolism, it is expected that they play key roles in neuronal development, which is not only complex but also a high energy process that includes the extension, patterning, and connectivity of neuronal processes^16^. Increased trafficking of lysosomes to dendrites and spines during development and in response to synaptic activity has been reported before^60,61^, supporting that lysosomal activity plays a role in dendritic differentiation. In this study, we found that during healthy conditions (WT) the volume of LAMP1^+^ lysosomes in developing dendrites was higher at the early stages of development and decreased as they reached maturity. In *Npc1*^*nmf164*^ PCs, the levels of dendritic LAMP1^+^ lysosomes were lower than WT at P14 and P30, but increased to WT levels at P21 suggesting that the aberrant activation of TFEB in these mice was driving the short-term increase of dendritic LAMP1^+^ lysosomes in NPC. Although the levels of LAMP1^+^ lysosomes and PDE^+^ mitochondria in *Pten-*cHet PC dendrites were significantly reduced at P14, the levels of dendritic mitochondria at P30 were remarkably higher than in WT. It is likely that after the fast overgrowth of dendrites at P14 in *Pten-*cHet PCs, the biogenesis of mitochondria was activated by energy (ATP) depletion^62^ and/or mitophagy activity was decreased due to the lack of PTEN^63^. However, contrary to *Npc1*^nmf164^ mice, not only were *Pten-*cHet mice able to increase the levels of mitochondria in PC dendrites with maturity, but also, they were able to prevent a significant atrophy of the dendritic trees. Additionally, contrary to *Npc1*^nmf164^ mice, *Pten-*cHet mice exhibited significant inhibition of TFEB catabolic signaling in PCs, suggesting that lack of catabolic signaling prevents the dramatic regression of PC dendritic trees. Because of the developmental dendritic deficits in PCs, both mutant mouse models showed early motor deficits in the horizontal ladder rung walking test. These results indicate that developing PC dendrites in *Npc1*^nmf164^ mice are suffering from noteworthy metabolic deficits that lead to the aberrant and simultaneous overactivation of anabolic and catabolic pathways. Furthermore, the increased engulfment of these structures by microglia suggests that metabolic deficits in these synaptic structures lead to their inability to establish functional synapses with PF, making these weak/non-functional synapses a target for pruning by microglia. Engulfment of synaptic inputs during developmental synapse elimination in healthy mice is driven by the lack of synaptic activity, where the weaker synapses are preferentially engulfed by microglia^25^. Overall, this study shows for the first time that NPC1-deficient mice have remarkable lysosomal-metabolic signaling deficits in developmental PCs that precede and predispose these cells to early degeneration in NPC. In conclusion, our studies highlight the importance of elucidating the role of metabolic balance in the differentiation, expansion, and patterning of neuronal dendrites and how disruption of these pathways leads to neuronal dysfunction and degeneration.

## Methods

### Animals

All experiments involving mice were conducted in accordance with policies and procedures described in the Guide for the Care and Use of Laboratory Animals of the National Institutes of Health and were approved by the Animal Care and Use Committees at the Rowan University School of Osteopathic Medicine and Providence College. The results and experiments of this study that involves animals are also reported in accordance with ARRIVE guidelines. The C57BL/6J-*Npc1*^*nmf164*^/J mouse strain (Jax stock number 004817) was provided by Dr. Robert Burgess at The Jackson Laboratory. *Npc1*^*nmf164*^ heterozygous mice were bred and housed in a 12/12-h light/dark cycle to generate both WT and *Npc1*^*nmf164*^ homozygous mutant mice. To produce NPC1-deficient mice with PCs expressing GFP (*Npc1*^*nmf164*^*-Pcp2*^*EGF*^), *Npc1*^*nmf164*^ heterozygous mice were intercrossed with the B6;FVB-Tg(Pcp2-EGFP)2Yuza/J (Jax stock number 004690). To study changes in mouse PCs dendrites during postnatal development caused by haploinsufficiency of *Pten,* the *Pten*^*flox*^ mouse strain (B6.129S4-*Pten*^*tm1Hwu*^/J, Jackson stock number 006440) was crossed to the *Pcp2-cre* strain (B6.129-Tg(Pcp2-cre)2Mpin/J, Jackson stock number 004146) to generate F1 heterozygous mice for both *Pten*^*f*lox/-^/Pcp2-Cre^+/-^ (here will be referred as *Pten-*cHet). Both males and females were used in this study, at a ratio of 2:2 when 4 mice were used. The tissue and results from WT mice used for the NPC1 deficiency studies were also used as a control for the experiments with *Pten*-cHet mice.

### Golgi-Cox staining technique

The Golgi-Cox staining technique was performed using and following the instructions of a commercially available kit (FD Rapid GolgiStain™ Kit, FD NeuroTechnologies Inc.) Briefly, after mice were euthanized with CO_2_, brains were dissected, immersed, and incubated in the impregnating solution (mixed solution A and B) for two weeks. After the two weeks of incubation, the impregnated solution was replaced by the 30% sucrose solution (solution C) and incubated for 72 h. Then, brains were removed from the solution, quick-froze, and stored at – 80 °C. For tissue sectioning, the brain was immersed and frozen in optimal compound temperature (OCT) media. Cryostat sections of 160 µm were collected in solution C, then rinsed in distilled water prior to being immersed in the developing solution (solutions D and E). After rinsing the tissue slices in distilled water, they were mounted onto slides, dried, and dehydrated using 75%, 85%, and 100% ethanol prior to being immersed in Histo-Clear II (National Diagnostics). Slides were mounted using Permount and allowed to dry overnight prior to analysis.

### Mouse perfusion and tissue preparation

After mice were euthanized with CO_2_, transcardial perfusion with 1× PBS followed by 4% paraformaldehyde was performed. Brains were carefully dissected and fixed by immersion in 4% paraformaldehyde overnight. After fixation, brains were rinsed in 1× PBS, immersed in 30% sucrose/PBS solution overnight at 4 °C, frozen in OCT, and cryosectioned as 40 μm and 50 μm floating sections.

### Immunohistochemistry

For immunofluorescence experiments, 40–50 μm floating sections were collected in 1× PBS, then rinsed once in 1× PBT (PBS + 1% Triton 100×), and incubated overnight at 4 °C in a cocktail of primary antibodies that were diluted in 1× PBT + 20% normal donkey serum. After the overnight incubation, sections were rinsed three times with 1× PBT for 10 min and incubated for 1.5 h in the corresponding secondary antibodies (1:500, Jackson-ImmunoResearch or Invitrogen). Cerebellar sections were then washed three times with 1× PBT for 10–15 min, incubated with DAPI, and mounted in Poly-aquamount (Polysciences). The following primary antibodies were used: rabbit anti-IBA1 (1:200, Wako, # 019-19741), mouse anti-CALB (calbindin, 1:200, Sigma-Aldrich, #C9848), rat anti-CD68 (1:200, Bio-Rad, #MCA1957), Lycopersicon esculentum(Tomato)-Lectin (1:200, Sigma-Aldrich, #L0401), guinea-pig anti-VGLUT1 (1:800, Synaptic Systems, #135404), rabbit anti-phosphorylated S6R (1:200, Cell Signaling, #2211), rabbit anti-phosphorylated AKT (1:200, Cell Signaling, #8112), rabbit anti-TFEB (1:200, Bethyl Laboratories, A303-673A), rabbit anti-FASN (1:200, Cell Signaling, #3180), rabbit anti-pyruvate dehydrogenase E 1 alpha (PDE) (1:200, GeneTex, # GTX104015), rat anti-CD107a (LAMP-1) (1:200, BioLegend, # 121602).

### Microscopy image analysis

For all the image analyses described below, investigators were blind to the genotype of the mice. PCs stained by the Golgi-Cox technique were imaged with the Keyence BZ-X800 imaging system using the 40× objective and the Quick Full Focus tool. To measure the total length and perform the Sholl-Analysis of these Golgi-Cox-stained PCs, the Simple Neurite Tracer plugin from the ImageJ software was used.

For the quantification of GFP^+^ spines that were colocalizing or not with VGLUT1^+^ presynaptic inputs from parallel fibers, a Nikon A1R Confocal System equipped with Live Cell 6 Laser Line and Resonant Dual Scanner was used to take 0.8 μm images with a 63× objective. To increase the visual magnification of these structures and facilitate the manual quantification of them, high magnified snapshots of the confocal images were taken using the Bitplane Imaris™ software (Oxford Instruments). Then, these snapshots with scale bars (see images in Fig. 1e) were used to measure the length of dendritic processes and manually count (Cell counter plugin) the number of spines per μm using the ImageJ software. Two to three images were taken per mouse (n = 4 mice).

For 3D image reconstructions and analyses, three sagittal 50 μm cerebellar sections were immunoreactive by free-floating immunohistochemistry. All the images analyzed by the Bitplane Imaris™ software were acquired using the Nikon A1R Confocal System. Confocal image stacks were acquired using a 40× objective lens with a 1 μm interval through a 40 μm z-depth of the tissue. Two to three confocal images per mouse were taken in the cerebellar cortex from the first 4 lobes (anterior region of the cerebellum). To quantify the total length and terminal points of cerebellar capillaries in the cerebellar cortex of postnatally developing mice, the Filament Tracer plugin from the Imaris™ software was used. Two to three images per mouse (n = 4 mice) were used for the quantifications. Quantitative analysis of 3D images to determine the volume of GFP or VGLUT1 inside microglia was performed using the Imaris™ Surface rendering tool. First, IBA1^+^ microglia were segregated using the Surface rendering tool. Then to quantify GFP from PC dendrites or VGLUT1 synaptic terminals contacted or engulfed by microglia, the “Mask all” tool was used to create a new channel of the GFP^+^ or VGLUT1^+^ areas inside of the created surface (in this case IBA1 surface) by clearing all the fluorescence that was not found overlapping/contacting the IBA1 rendering surface. The sum of the GFP or VGLUT1 volume contacted or inside the IBA1 surface was calculated and provided by the software then used for the data analysis presented here. Levels of VGLUT1 immunofluorescence in the molecular layer of cerebellar sections were measured in confocal images using the Surface rendering tool, where a region of interest of 360 × 460 pixels was selected and the total volume of VGLUT1 fluorescence was calculated by the software.

Quantifications of the PDE and LAMP1 total volume inside CALB^+^ dendrites were performed by cropping a region in the ML (300 μm height × 400 μm wide) in 40× confocal images and creating a 3D surface rendering for CALB that was used to obtain the sum of the volume of the CALB^+^ dendrites. Then, the “Mask all” tool was used to create a new channel for the PDE^+^ or LAMP1^+^ areas inside of the created surface (in this case CALB surface) by clearing all the fluorescence that is not found overlapping/contacting the rendering surface. 3D surface renderings were created for the newly created PDE and LAMP1 channels in order to determine the volume of this staining that was inside the CALB^+^ dendrites. The Imaris™ software calculated and provided the measurements of the respective volumes for PDE and LAMP1, and the percentage of these markers in PC dendrites were calculated by dividing them by the CALB volume of the dendrites.

The confocal images and Imaris™ surface rendering tool were also used to measure the levels of pS6R specifically in PC dendrites and the pAKT volume in PC soma. The dendritic pS6R immunoreactivity in dendrites was confirmed by colocalization with CALB. FASN fluorescence raw intensity per PC soma was measured using ImageJ, where the soma of the cells was manually traced to select the region of interest for the measurement. The intensity value was then divided by the area of the traced cell. PCs with nuclear labeling of TFEB were manually counted after selecting a region of interest using a box of 50 × 40 pixels and divided by the total number of PCs inside the box to calculate the percentage of PCs with TFEB nuclear labeling. NeuroTrace was used to label the PC soma when FASN and TFEB antibodies were used.

### Electron microscopy

For electron microscopy, WT and *Npc1*^*nmf164*^ mice (12 weeks of age) were perfused with 4% PFA, then brains were dissected and hemisected in the midsagittal plane. One of the hemisections was fixed overnight (2% paraformaldehyde + 2% glutaraldehyde diluted in 0.1 M cacodylate buffer with 0.05% CaCl2) for electron microscopy. The process of resin embedding was performed as previously described^7,64^. Briefly, small pieces of the processed cerebella were infiltrated with 50/50 Epon-Araldite resin and propylene oxide for 1 h, then in 100% Epon-Araldite and left in the desiccator overnight. The next day the cerebellum samples were placed in cubic molds and embedded in 100% resin. The resin block was trimmed and, using an ultramicrotome (Sorvall MT-2), longitudinal sections were cut; semi-thin sections (1 μm thick) for light microscopy, and ultrathin (90 nm) for electron microscopy. For light microscopy, semi-thin sections were stained using methylene blue-azure II and basic fuchsin. Thin sections were examined with a JEOL JEM-1011 electron microscope equipped with a Gatan digital camera (Model-832) to describe the ultrastructural features of the cerebellar ML.

### Ladder rung walking task

The apparatus consisted of 100 metal rungs (3 mm each) forming a ladder floor that is raised above the surface (from Maze Engineers). The minimum space between the metal rungs is 1 cm. However, for this study, to challenge the P30 mice, 11 metal rungs were removed in the center of the apparatus creating a space of 2 cm between the rungs in the central area. Therefore, mice start and finish the test walking over 1cmm separated rungs but encountering a 22 cm central stretch with 2 cm space between the metal rungs. Each mouse was placed at one end of the apparatus (one at a time) and videotaped from the side as the mouse walks-transversely across the ladder from the beginning to the end. Investigators recording the video were blinded to the genotype of the mice. Two investigators blinded to the genotype of the mice, quantified misses and slips of the forelimbs or hindlimbs for each mouse between the stretch that contain the 2 cm separation. The test was repeated three times and results between trials 1 and 3 were compared.

### Western Blot

Protein samples were extracted with RIPA buffer, separated by SDS-PAGE gel electrophoresis and transferred to nitrocellulose or PVDF membrane. Before incubation with primary antibodies, membranes were blocked in Intercept^®^ (PBS) Protein-Free blocking buffer (LI-COR), and after primaries antibody incubation (rabbit anti-pyruvate dehydrogenase E 1 alpha (PDE), 1:2000, GeneTex, # GTX104015; and mouse anti-GAPDH, 1:5000, Sigma Aldrich, # G8795), the appropriate fluorescent-conjugated antibody (LI-COR) was used as a secondary antibody. For detection and bands analysis, membranes were imaged and analyzed using the iBright Imaging System (Thermo Fisher Scientific).

### Statistical analysis

Data were analyzed using GraphPad Prism software. Significance was calculated using unpaired t-tests for comparisons between two groups. p-values are provided as stated by GraphPad Prism software and significance was determined with p-values less than 0.05.

## Supplementary Information

Supplementary Figures.

## Data Availability

All data generated or analyzed during this study are included in this manuscript and its Supplementary Information files.
