# Supplementary material for: Disruptive lysosomal-metabolic signaling and neurodevelopmental deficits that precede Purkinje cell loss in a mouse model of Niemann-Pick Type-C disease
Source: Sci Rep. 2023 Apr 6;13:5665. doi: 10.1038/s41598-023-32971-0 (PMC10079843; doi:10.1038/s41598-023-32971-0)
Supplement: Supplementary file 1 — Supplementary Figures. [file 41598_2023_32971_MOESM1_ESM.pdf]

Supplementary Information

Disruptive lysosomal-metabolic signaling and neurodevelopmental deficits that precede Purkinje cell loss in a mouse model of Niemann-Pick Type-C disease

Sarah Kim[2], Kathleen Ochoa[2], Sierra E. Melli[2], Fawad A. K. Yousufzai[2], Zerian D. Barrera[4], Aela A. Williams[3], Gianna McIntyre[2], Esteban Delgado[2], James N. Bolish[3], Collin Macleod[1], Mary Boghos[1], Hayden Lens[1], Alex Ramos[1], Vincent B. Wilson[4], Kelly Maloney[2], Zachary M. Padron[2], Amaal H. Khan[2], Rosa E. Blanco[5], Ileana Soto[1*]

Supplementary Figures:

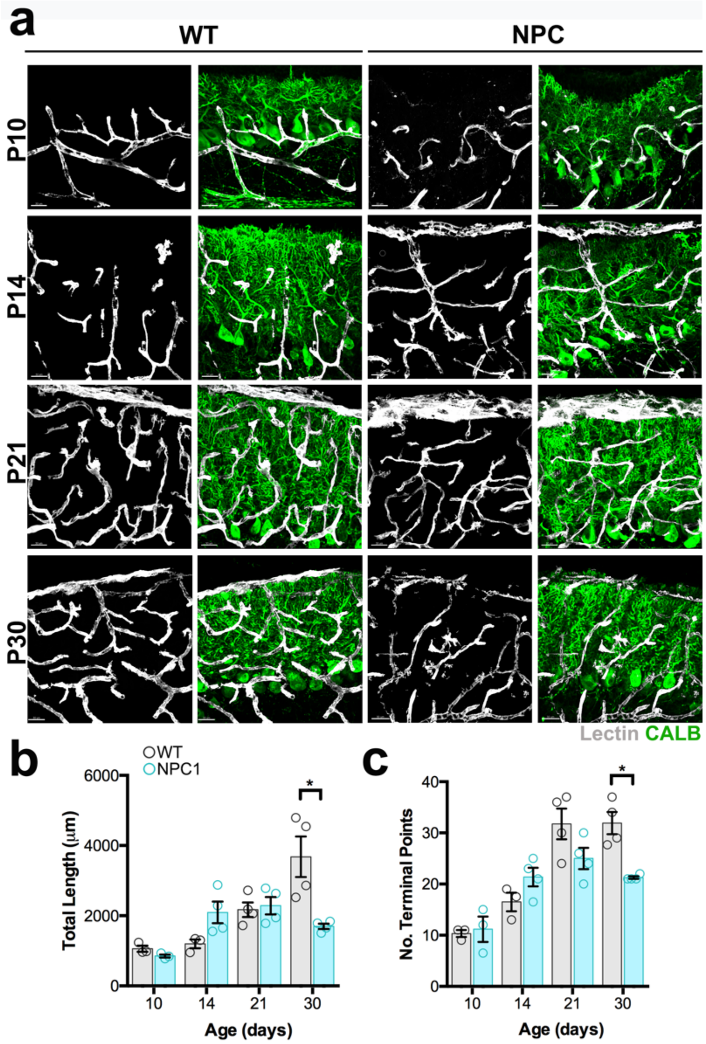

Fig.S1. a) Molecular layer Tomato-Lectin labeled capillaries at different stages of postnatal development in WT and NPC1 mice. b) Quantitative analysis of capillary Total Length in the molecular layer of WT and NPC1 mice. c) Quantitative analysis of the number of Terminal Points in capillaries from the molecular layer of WT and NPC1 mice. Data are presented as mean ± SEM, n= 2 images from 4 mice/genotype/age. *P < 0.05. Scale bars: (a) 30 μm.

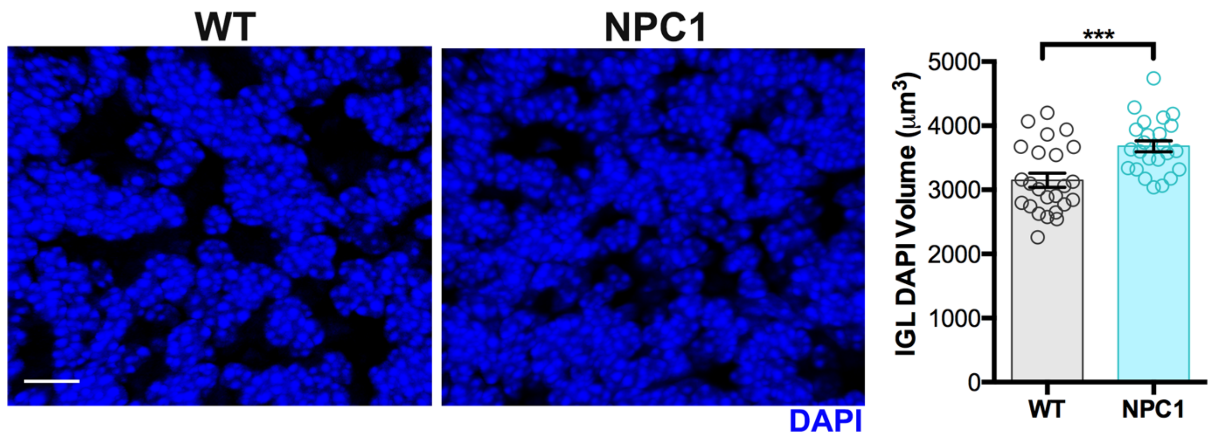

Fig. S2. Total volume of DAPI staining in the inner granule layer (IGL) of P30 WT and *Npc1*^{nmf164} using the Imaris surface rendering tool. Data are presented as mean ± SEM, n= 2 images from 4 mice/genotype/age. ***P < 0.001. Scale bars: (a) 10 μm.

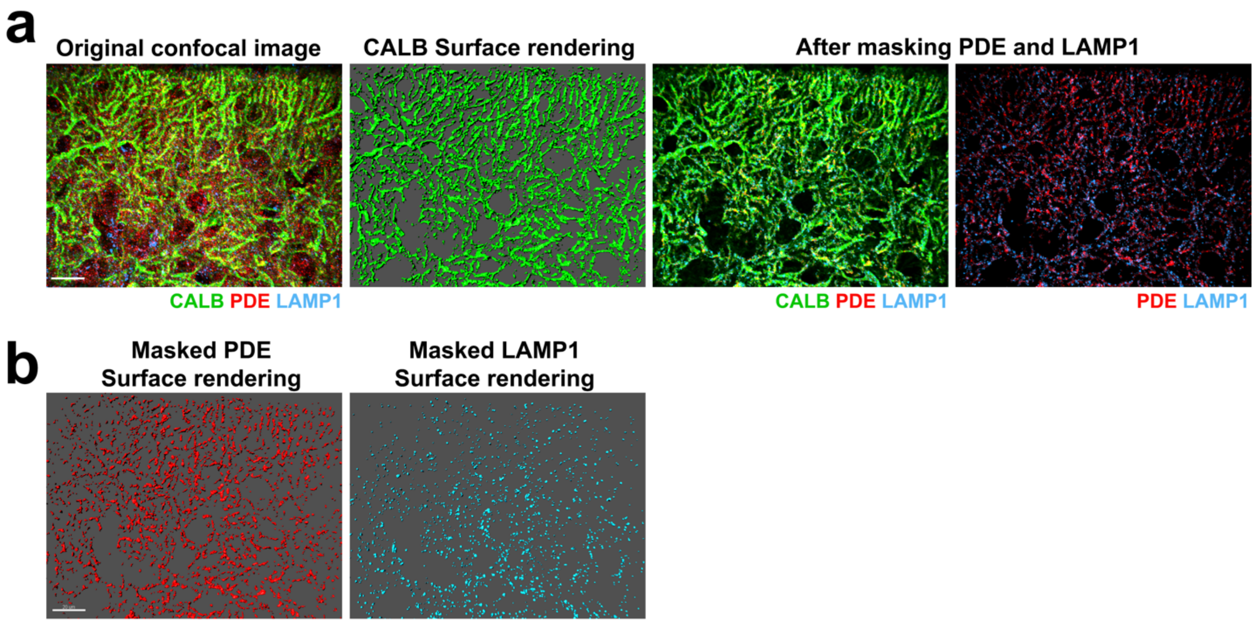

Fig. S3. a) To segregate the PDE and LAMP1 immunostaining in PC dendrites from the immunoreactivity in other cellular structures, 3D confocal images with CALB, PDE, and LAMP1 immunoreactivity were used (original confocal image). A surface rendering of CALB was generated and used to mask the PDE and LAMP1 inside the CALB surface. b) Surface renderings were generated from the masked PDE and LAMP1 for quantitative analysis. Scale bars: (a) and (b) 20 μm.

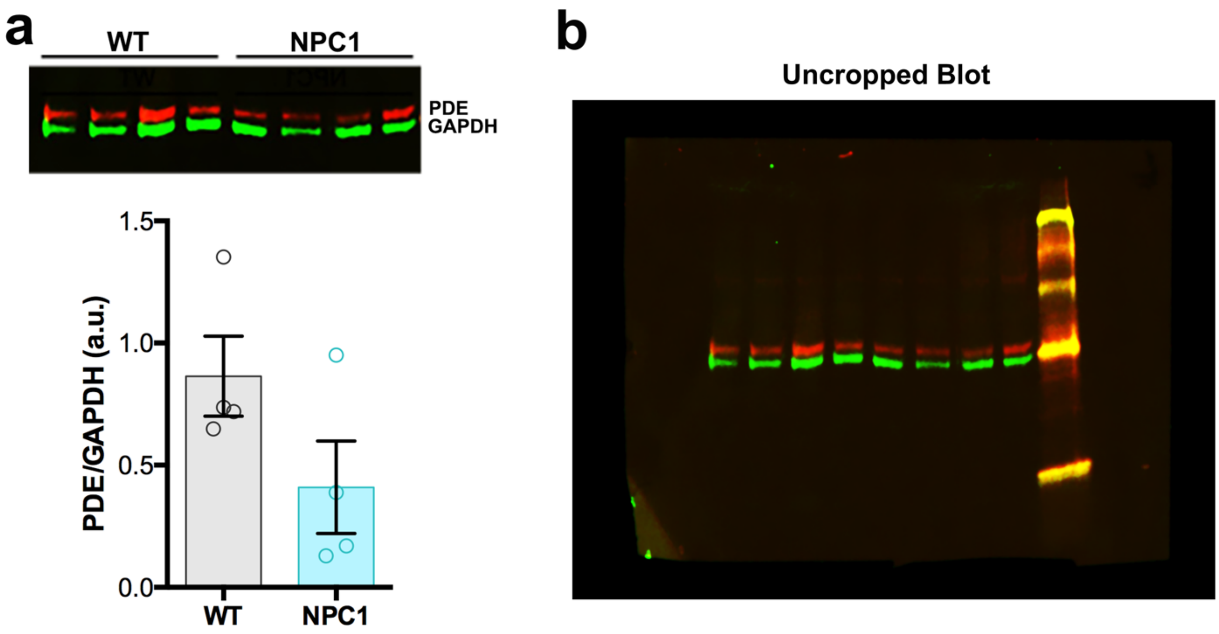

Fig. S4. a) PDE1A protein levels in WT (n=4) and *Npc1*^{nmf164} (n=4) cerebella as measured by Western Blot. GAPDH protein levels are used as loading control. Data are presented as mean ± SEM. b) Original image of the blot before cropping.

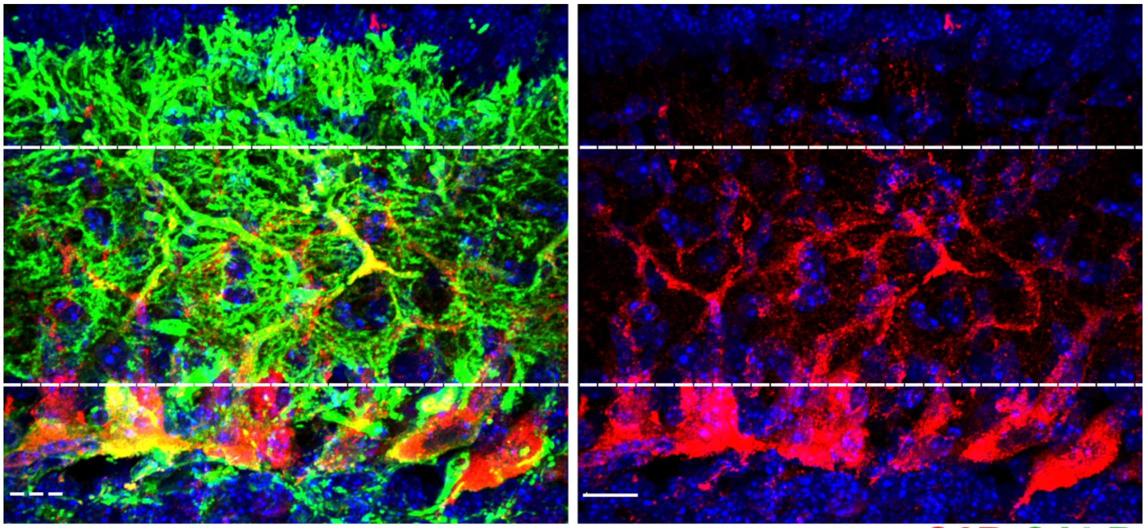

Fig. S5. Co-immunoreactivity of pS6R and Calbindin in a P14 *Npc1*^{nmf164} cerebellar section showing colocalization of ML pS6R immunoreactivity with Calbindin^{+} PC dendrites (between dotted lines). Quantitative analyses sowed in Fig. 5 were specific for this region. Scale bar: 20μm.

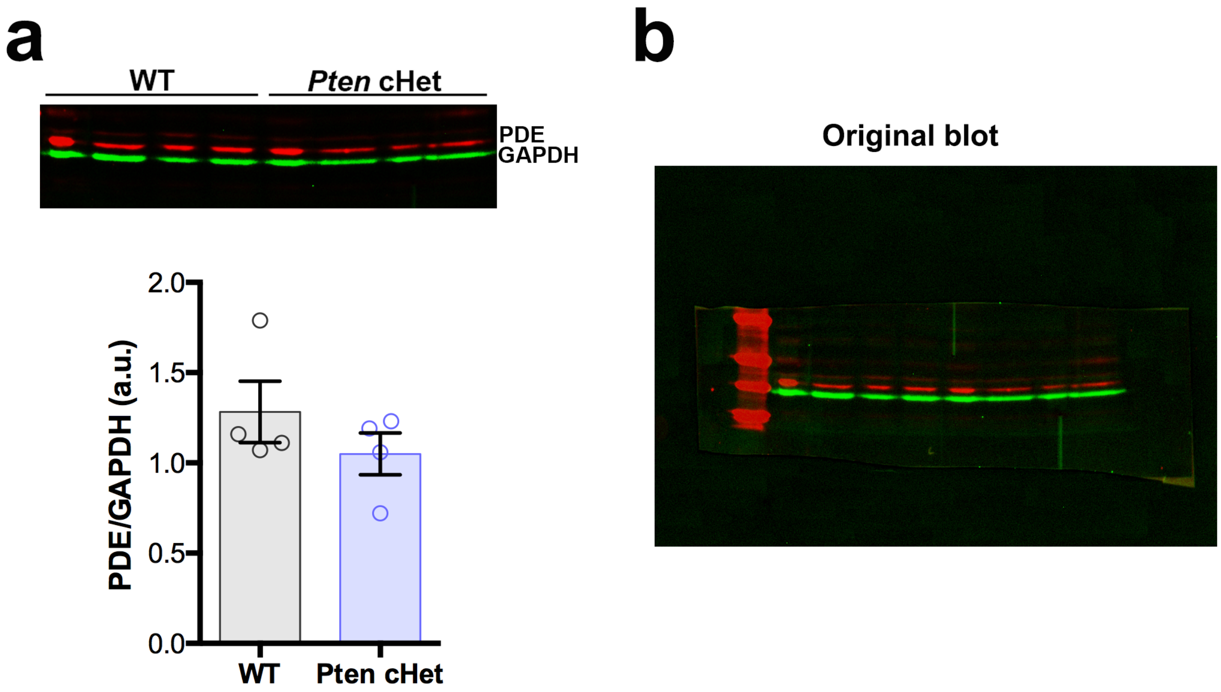

Fig. S6. a) PDE1A protein levels in WT (n=4) and *Pten* cHet (n=4) cerebella as measured by Western Blot. GAPDH protein levels are used as loading control. Data are presented as mean ± SEM. b) Original image of the blot, which was cropped to keep specifically the region that contains the bands of interest and to reduce the amount of antibody needed for the immunoreaction.
